# Roles of monocyte chemotactic protein 1 and nuclear factor-κB in immune response to spinal tuberculosis in a New Zealand white rabbit model

**DOI:** 10.1590/1414-431X20165625

**Published:** 2017-02-20

**Authors:** X.H. Guo, Z. Bai, B. Qiang, F.H. Bu, N. Zhao

**Affiliations:** 1 The Third Department of Orthopedics, the Fifth Hospital of Harbin, Harbin, China; 2 Operating Room, the Fifth Hospital of Harbin, Harbin, China

**Keywords:** Monocyte chemotactic protein 1, Nuclear factor-κB, Spinal tuberculosis, Immune response

## Abstract

This study aimed to explore the roles of monocyte chemotactic protein 1 (MCP-1) and nuclear factor kappa B (NF-κB) in immune response to spinal tuberculosis in a New Zealand white rabbit model. Forty-eight New Zealand white rabbits were collected and divided into four groups: experimental group (n=30, spinal tuberculosis model was established), the sham group (n=15, sham operation was performed) and the blank group (n=3). The qRT-PCR assay and western blotting were applied to detect the mRNA and protein expressions of MCP-1 and NF-κB in peripheral blood. ELISA was used to measure serum levels of MCP-1, NF-κB, IFN-γ, IL-2, IL-4, and IL-10. Flow cytometry was adopted to assess the distributions of CD4^+^, CD8^+^ lymphocytes and CD4+ CD25+ Foxp3 lymphocyte subsets. Compared with the sham and blank groups, the mRNA and protein expressions of MCP-1 and NF-κB in the experimental group were significantly increased. The experimental group had lower serum levels of IL-2 and IFN-γ and higher serum level of IL-10 than the sham and blank groups. In comparison to the sham and blank groups, CD4^+^ T lymphocyte subsets percentage, CD4^+^/CD8^+^ ratio and CD4^+^ CD25^+^ Foxp3+ Tregs subsets accounting for CD4^+^ lymphocyte in the experimental group were lower, while percentage of CD8^+^ T lymphocyte subsets was higher. Our study provided evidence that higher expression of MCP-1 and NF-κB may be associated with decreased immune function of spinal tuberculosis, which can provide a new treatment direction for spinal tuberculosis.

## Introduction

Spinal tuberculosis, a destructive form of tuberculosis, is very common in young adults and children, it accounts for approximately fifty percent of all cases of musculoskeletal tuberculosis, and its incidence is increasing in developed nations (1). The World Health Organization reported 8.8 million new tuberculosis cases in the world in 2010 (2). In China, bone and joint tuberculosis are the most common secondary tuberculosis, of which more than 50% are spinal tuberculosis. About 5% of patients will develop a severe kyphotic deformity resulting in spinal cord compression, pain, costopelvic impingement, cosmetic concerns and cardiopulmonary dysfunction (3). Changes in the immune system are closely related to the occurrence, development and outcome of tuberculosis, and T cells such as CD4^+^ and CD8^+^, which are related to cellular immunity, play important roles. A variety of cytokines released among immune cells can have important impacts on the immune response (4). Therefore, understanding the effect of tuberculosis immune factors is a new direction for studying the treatment of spinal tuberculosis.

Monocyte chemotactic protein 1 (MCP-1) is a member of the chemokine cytokine CC subfamily. It is mainly produced by fibroblasts, endothelial cells, white blood cells and mononuclear cells (5). MCP-1 can also play an important role on the chemotaxis and activation of lymphocytes and mononuclear cells (6). MCP-1 is a key mediator in the inflammatory process, as its signaling induces the production of numerous proinflammatory cytokines. MCP-1 is one of the important cytokines regulating immune response (7). Nuclear factor kappa B (NF-κB), a transcription factor, is widely involved in the regulation of many genes in eukaryotic cells, and has a significant role in the immune response (8). NF-κB can be activated by inflammatory cytokines and other stimulants, which can regulate the gene expression of cytokines, growth factors, chemokines, and adhesion molecules. Thereby, it affects the body’s acquired or innate immune response, inflammatory reaction, cell differentiation, apoptosis and a variety of other biological functions (9–11). The relationship between MCP-1 polymorphism and susceptibility to spinal tuberculosis, or between serum levels of MCP-1 and postoperative recurrence of spinal tuberculosis, have been reported in previous studies (12,13). However, there are few studies on the role of MCP-1 and NF-κB in the immune response of spinal tuberculosis. Therefore, this study aimed to explore the roles of MCP-1 and NF-κB in immune response to spinal tuberculosis by constructing a New Zealand rabbit model of spinal tuberculosis, and provide a new target for the treatment of spinal tuberculosis.

## Material and Methods

### Ethics statement

This study was approved by the Ethics Committee of the Fifth Hospital of Harbin (No. 201510117). All procedures were in compliance with ethical guidelines for animal experiments.

### Animals

A total of 48 male New Zealand white rabbits (aged 18∼22 weeks with an average weight of 2.4±0.4 kg) were provided by the animal experimental center of the Fifth Hospital of Harbin. Animals without trauma, infection, bone deformity, or other systemic diseases, were raised in standard feeding cages at the constant temperature of 25°C, under a 12 h light/dark cycle. All rabbits were fed standard rabbit chow and had free access to drinking water.

### Establishment of a rabbit model of spinal tuberculosis

Animals were randomly divided into 3 groups: experimental group (n=30), in which the spinal tuberculosis model was established; sham-operated group (n=15); and blank group (n=3), in which rabbits were kept under the same conditions but without any treatment. The infection strains were H37Rv standard strain (obtained from the Fifth Hospital of Harbin, China), and the culture medium was produced by Roche (Jinan Cell Biology, China). After 2 weeks, the colony with good growth was harvested for the preparation of 1 mg/mL bacterial suspension. For the surgical procedure, rabbits were anesthetized, had the hair removed, were disinfected, and the 3rd–5th lumbar vertebrae were exposed from the left side of the peritoneum. Perforations of 0.5×0.8 cm were drilled in the upper part of the L4 vertebra near the intervertebral disc. In the experimental group, the perforations were filled with a gelatin sponge containing 1.0 mL of bacteria suspension. In the sham group, the perforations received a gelatin sponge soaked in sterile saline. During surgery, the principles of asepsis, standard prevention, and animal ethics were strictly followed. After surgery, the rabbits were kept in separate cages, and their wound healing condition, everyday activity, mental health, eating and drinking conditions were monitored.

After 8 weeks, peripheral venous blood was collected for analysis. After anesthetizing, an incision was performed and tissue samples of the 3–5 lumbar vertebra were harvested for *Mycobacterium tuberculosis* culture and later hematoxylin-eosin (HE) staining. Pathological sections and photographs were blindly analyzed. If animals from the experimental group were positive for tuberculosis culturing, or if there was emergence of pathological changes characteristic of spinal tuberculosis, the success of the model was determined. Then, the experimental rabbits were euthanized and carcasses were sealed in plastic bags and sent to the hospital facility for incineration. Ultraviolet radiation was used to disinfect the animal’s breeding sites for 24 h.

### Quantitative real-time polymerase chain reaction (qRT-PCR)

Two mL of EDTA-K2 anticoagulant (Ming Xiu Biotechnology Co., Ltd., China) was added to lymphocyte separation solution (Tian Jin Hao Yang Biological Manufacture Co., Ltd., China) to isolate mononuclear cells, and Trizol (Invitrogen, USA) was used to extract the total RNA. RNA absorbance at 260/280 was detected with an ultraviolet spectrophotometer to determine total RNA concentration and purity. The extracted RNA was preserved at –80°C until use. PrimeScript™ RT reagent Kit (Perfect Real Time; TaKaRa, China) was used for the reverse transcription from total RNA to cDNA, which was preserved at –20°C for further use. β-actin was used for reference and qRT-PCR was performed with the ABI7500 quantitative PCR (ABI, USA). The primer sequences are shown in Table 1, and reaction conditions were as follows: denaturation at 95°C for 5 min, denaturation at 94°C for 30 s, annealing at 55°C for 30 s, extension at 72°C for 30 s, for a total of 30 cycles. PCR results were analyzed with Opticon Monitor 3 (BioRad, USA). The lowest point of each log expansion curve parallel was selected for the threshold (Threshold cycle, Ct), and the 2^-ΔΔCt^ method was used to calculate the relationships between the expression levels of the experimental and control group. The formula was as follows: ΔΔCT=ΔCt_experimental group_ - ΔCt_control group_, and ΔCt=Ct_target gene_-Ct_β-actin_. The experiment was repeated 3 times (14).

**Table:**
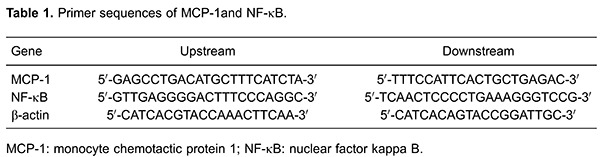

### Western blotting

Lymphocyte separation medium was used to isolate and collect lymphocytes. Cell lysis solution with protease inhibitor (RD Company, USA) was added to iced cell lysis. The ultrasonic suspension was then centrifuged at 10,800 *g* for 10 min at of 4°C. Supernatant plus 5× protein sample buffer was taken and boiled for 10 min. MCP-1 sample, which had been processed and detected, was frozen at –20°C for storage. After the cell count was done, the cell suspension liquid was extracted according to the specification of the protein preparation kit (GENMED Scientifics, USA). Extracted nuclear proteins plus 5× protein sample buffer was taken and boiled for 10 min. The processed NF-κB sample was frozen at -20°C for storage. A total of 10% polyacrylamide gel electrophoresis was adopted to isolate protein. The protein was then transferred to polyvinylidene fluoride and 5% skim milk was used to seal for 2 h. After membrane cleaning, the samples were put into 1:1000 diluted MCP-1 antibody, NF-κB antibody and β-actin (Abcam, USA) and incubated overnight at 4°C for first antibody detection. Membranes were cleaned again, then samples were put into 1:1000 diluted for second antibody detection at 37°C for 2 h. Membranes were washed a third time and prepared for electrochemiluminescence (ECL) development.

### Enzyme-linked immunosorbent assay (ELISA)

Three mL of peripheral venous blood was collected without anticoagulant, serum was separated and frozen at –70°C until use. For analysis, the serum was thawed in a 37°C water bath. MCP-1, IFN-γ, IL-4, IL-2 and IL-10 kits (Shenzhen Jingmei Biological Engineering Co., Ltd., China) were counterbalanced at room temperature for 20 min. Standard wells were filled with different concentrations of 50 µL of the standard samples, and 5 dilutions of 50 µL sample to be tested were added to the sample wells. Blank wells had nothing added. Fifty microliters of enzyme-labeled reagent were added to the standard and sample wells. The wells were sealed and kept at 37°C for 60 min. The plate was washed 5 times and then dried. A and B color agents (50 µL each) were added to each well, which was maintained in the dark at 37°C for 15 min. Stop buffer (50 µL) was then added to each well, and absorbance was measured at 450 nm within 15 min. The standard curve was drawn and the corresponding concentration was determined from the absorbance value of the sample, and then multiplied by the total dilution, which was the actual concentration of the sample.

Peripheral venous blood was collected and preserved. Lymphocyte separation medium was used to isolate and collect lymphocytes. Cells were counted and 5×10^6^ cells were removed into a 15 mL conical centrifuge tube. Nucleoproteins were extracted and kept at –70°C according to the specification of the cell soluble total nuclear protein preparation kit (GENMED SCIENTIFICS Company, USA). The nucleoproteins precipitate was then removed from the –70°C refrigerator and thawed in a water bath. MCP-1, IFN-γ, IL-4, IL-2 and IL-10 kits (Shenzhen Jingmei Biological Engineering Co., Ltd. Shenzhen, China) were counterbalanced at room temperature for 20 min. Procedures to detect the content of NF-κB p65 in the nuclear extract were carried out in strict accordance with the kit instructions.

### Flow cytometry

A total of 100 µL of fresh samples were added into flow-type sample tube 1 and tube 2. Twenty µL mouse anti-rabbit CD4-FITC/CD8-PE/CD3-PCS mixed antibody (Beckman Coulter, USA) was added to tube 1. Twenty µL of the same type of control antibody was added to tube 2, incubated at 4°C for 30 min in the dark and then washed with phosphate buffered saline (PBS) buffer. The supernatant was removed and this step was repeated 2 times, centrifuged at 60 *g* for 10 min at 4°C. The red cell lysis solution was added to tubes 1 and 2 (each tube 300 µL). Light was avoided for 20 min at 4°C, washing was repeated 2 times, and PBS 200 µL cell liquid suspension was added. Tube 2 was used as a negative control to adjust the parameters, and tube 1 was used for detection. Lymphocyte gating was selected according to cell size. Next, using the CD3+ cells as gating, the percentage of CD4+, CD8+ T lymphocyte subsets could be obtained. The distribution of CD4+ CD25+ Foxp3+ lymphocyte subsets was detected using mouse anti rabbit CD4-FITC/CD25-PE antibody mix (Beckman Coulter), and the remainder of the procedure followed the above methods. The fluorescence parameters were obtained and analyzed with MultiSET software (Becton Dickinson Bio-sciences, USA).

### Statistical analysis

Data were analyzed with SPSS 22.0 statistical software, and are reported as means±SD. The *t*-test was applied for comparison between two groups and single factor variance analysis (one-way ANOVA) was used for comparison among several groups. Enumeration data are reported as percentage and were analyzed with the chi-square test. Pearson correlation method was applied for correlation analysis. P<0.05 indicated statistical significance.

## Results

### Establishment of a rabbit model of spinal tuberculosis

**Figure 1:**
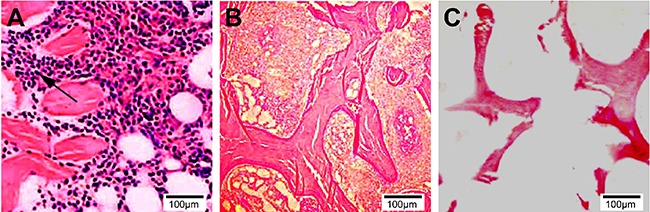
Hematoxylin-eosin staining of adjacent vertebral bone tissues of rabbits. *A*, Spinal tuberculosis experimental group; *B*, sham-operated group; *C*, blank group. Arrow: formation of necrosis abscess in the medullary cavity.

All rabbits had an uneventful recovery. Anatomy analysis was carried out under anesthesia after 8 weeks. The experimental group showed apparent bone destruction in the operated vertebra with a darker intervertebral disc annulus fibrosus and more concave endplate. The experimental group exhibited bone destruction in vertebral lesions, and structural disorder in bone trabeculae, surrounded by many invasive inflammatory cells (Figure 1). All specimens in the experimental group showed a positive tuberculosis culture except in two contaminated specimens. There were no positive cultures in the sham and blank groups after 6 weeks culture. There was a significant difference in the culture-positive specimens among the three groups (P<0.05).

### mRNA and protein expressions of MCP-1 and NF-κB

**Figure 2:**
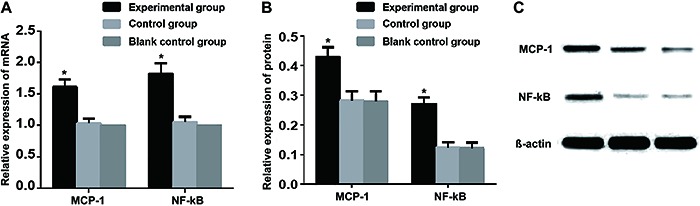
*A*, Relative mRNA expression (%) of monocyte chemotactic protein 1 (MCP-1) and nuclear factor kappa B (NF-κB) among the spinal tuberculosis (experimental), sham operated (control) and blank groups. *B*, Protein expressions (%) of MCP-1 and NF-κB among the same groups. *C*, Western blotting of the protein expressions of MCP-1 and NF-κB. *P<0.05 compared to the blank and sham groups (ANOVA).

The mRNA expression of MCP-1 in the experimental group was higher than in the sham and blank groups (P<0.05). Likewise, a higher expression of NF-κB mRNA was also detected in the experimental group than the other two groups (P<0.05). However, no significant difference was found in the mRNA expressions of MCP-1 and NF-κB between the sham and blank groups (P>0.05; Figure 2A).

The protein expressions, detected by western blotting, of MCP-1 and NF-κB in the experimental group were higher than those in the sham and blank groups (all P*<*0.05). No difference was found in the protein expressions of MCP-1 and NF-κB between the sham and blank groups (all P>0.05; Figure 2B and C).

### Serum MCP-1 and NF-κB levels

**Figure 3:**
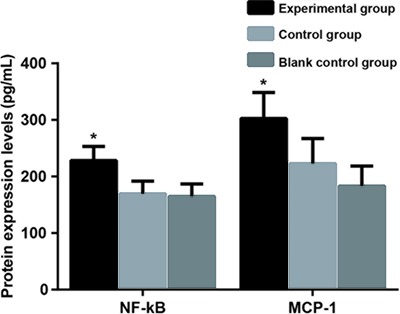
Serum monocyte chemotactic protein 1 (MCP-1) and nuclear factor kappa B (NF-κB) levels among the spinal tuberculosis (experimental), sham-operated (control) and blank groups detected by ELISA. *P<0.05 compared with the blank and sham groups (ANOVA).

ELISA indicated that serum MCP-1 and NF-κB levels in the experimental group were higher than those in the sham and blank groups (all P*<*0.05), while there was no significant difference between the sham and blank groups (P*>*0.05; Figure 3).

### Serum levels of IFN-γ, IL-2, IL-4 and IL-10

**Figure 4:**
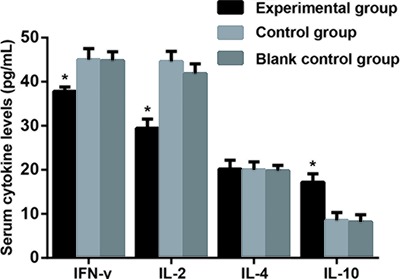
Serum levels of IFN-γ, IL-2, IL-4 and IL-10 among the spinal tuberculosis (experimental), sham-operated (control) and blank groups. *P<0.05 compared with the blank and sham groups (ANOVA).

As shown in Figure 4, serum IL-2 level in the experimental group was lower than the sham and blank groups (P*<*0.05). By contrast, no significant difference was found among the three groups in serum IL-4 levels (P=0.934). In addition, the experimental group presented a higher serum IL-10 level than the sham and blank groups (both P<0.05). However, the experimental group showed a lower serum level of IFN-γ than the sham and blank groups (both P<0.05). There was no statistical difference in serum levels of IFN-γ, IL-2, IL-4 and IL-10 between the sham and blank groups (all P>0.05).

### T-cell subset distribution

**Figure 5:**
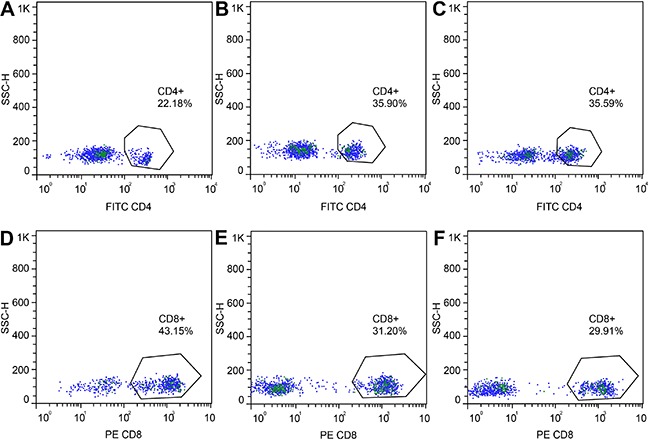
Percentages of CD4+ lymphocytes in the spinal tuberculosis experimental group (*A*), in the sham-operated group (*B)*, and in the blank group (*C*). Percentages of CD8+ lymphocytes in the experimental (*D)*, sham-operated (*E)*, and blank groups (*F*).

As shown in Figure 5 and Table 2, the percentage of CD4^+^T lymphocyte subsets was lower in the experimental group than in the sham and blank groups (all P<0.05). However, the percentage of CD8^+^T lymphocyte subsets in the experimental group was higher than that in the sham and blank groups (both P<0.05). A lower percentage of CD4^+^/CD8^+^ was found in the experimental group than in the sham and blank groups (both P<0.05). Meanwhile, the ratio of CD4^+^ CD25^+^ Foxp3^+^ Tregs subsets accounting for CD4^+^ lymphocytes in the experimental group was also lower than in the sham and blank groups (both P*<*0.05).

**Table:**
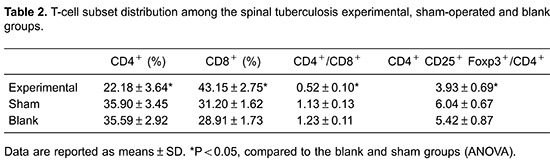

### Correlations of MCP-1 and NF-κB with immune function of rabbit with spinal tuberculosis

Correlation analysis (Table 3) showed a significant positive correlation between the MCP-1 and NF-κB with an r value of 0. 910 (P*<*0.05); Moreover, the expressions of MCP-1 and NF-κB were negatively correlated with the levels of serum IFN-γ and IL-2, as well as with the percentage of CD4^+^ T lymphocyte (all P*<*0.05), while positively correlated to the ratio of CD4^+^ CD25^+^ Foxp3^+^ Tregs subsets accounting for CD4^+^ lymphocytes and with the levels of IL-10 (both P<0.05). However, no significant correlation was found between MCP-1 and NF-κB expressions and the percentages of CD8^+^ T lymphocytes (both P>0.05).

**Table:**
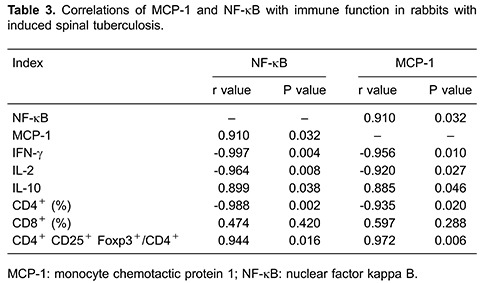

## Discussion

Spinal tuberculosis remains a growing public health issue, and the incidence of neurologic involvement is approximately 10 to 47% (15). If affecting the spinal joint, it can cause spinal nerve damage and deformity, with a high disability rate, and seriously affect the quality of life of patients (1). It is believed that the direct damage caused by a large number of *M. tuberculosis* in the tissue and the immune-mediated reactions are related to spinal tuberculosis (16). Therefore, understanding the role of the immune response factors involved in spinal tuberculosis will help find new treatment targets for the disease.

In this study, MCP-1 and NF-κB mRNA and protein expressions were found to be increased in the peripheral blood of rabbits, and the expression of MCP-1 was positively correlated with NF-κB in the spinal tuberculosis model. MCP-1 is a member of the CC chemokine family, an important macrophage/monocyte chemotactic factor and MCP-1 and plays an important role in immunity to spinal tuberculosis (17). The results of our research agree with a study that reported that the MCP-1 produced by immune cells extracted from patients with tuberculosis was significantly higher than those in healthy people (18). Under the action of oxidative stress, cytokines and endotoxins, NF-κB, as a cellular immune factor, shifts rapidly to the nucleus and starts the transcription of target genes. It is also involved in the regulation of cytokines, immune reaction and inflammatory reaction. Some inflammatory cytokines, transcription growth factor (TGF-β) and other factors can stimulate the inflammatory reaction of NF-κB (19,20). At the same time, there are reports that NF-κB can be a gene promoter or enhancer, containing NF-κΒ binding sites (21). NF-κB is another signaling molecule involved in cell activation and in amylin upregulation by the effect of MCP-1 (22). NF-κB can regulate MCP-1 and participate in the occurrence and development of many diseases, which is in accordance with the result of this study.

Furthermore, it was also found that MCP-1 and NF-κB expression levels were positively correlated with IL-10 levels in the peripheral blood of animals with tuberculosis, and with the percentage of CD4^+^ CD25^+^ Foxp3^+^ Tregs subsets accounting for CD4^+^ lymphocyte, while both of the expressions were negatively correlated with IFN-γ, IL-2 and the percentage of CD4^+^ T lymphocyte. CD4^+^ T cells can play significant roles in common autoimmune diseases such as rheumatoid arthritis and systemic lupus erythematosus (23). CD4^+^ T cells can be divided into two subsets, Th1 and Th2, the imbalance of which can lead to decreased immune function, thereby weakening the body's ability to resist infection (24). IFN-γ is the key factor to control the infection of tuberculosis (25). IL-2 is produced in activated CD4^+^ T cells, and can induce the proliferation and differentiation of T cells, promote the secretion of various cytokines and enhance the proliferation and the killing activity of immune cells (26). IL-2 is a type of Th2 cytokine, which has anti-inflammatory effects, and can inhibit the production of TNF-α and down-regulate the expression of the TNF receptor (27). Treg is a special type of immune suppressive cell with a variety of functional groups. CD4^+^ CD25^+^ Foxp3^+^ Treg is one of the most important subgroups (28). At the site of infection, high levels of TGF-β can promote the differentiation of Tregs, which can provoke positive costimulatory signals and activate related cytokines. This can result in an increased number of Tregs in the lesion, and the immune function of effector T cells are greatly inhibited (29).

In conclusion, this study confirmed that higher expressions of MCP-1 and NF-κB are associated with decreased immune function in spinal tuberculosis. This study can help to understand the immune mechanism of spinal tuberculosis, and provide new directions for treatment. However, due to the small number of animals and the short observation time, these results still need to be confirmed with animal experiments and clinical research. The mechanism of inflammatory injury in spinal tuberculosis should also be studied further.
